# Myofibroma of the pinna: a case report and review of the literature

**DOI:** 10.1186/s40792-024-01879-w

**Published:** 2024-04-23

**Authors:** Nupur Bhatt, Lydia Pan, Tom Ben-Dov, Scott Rickert

**Affiliations:** Division of Pediatric Otolaryngology, Department of Otolaryngology–Head & Neck Surgery, NYU Langone Health, 240 East 38th Street, Fourteenth Floor, New York, NY 10016 USA

**Keywords:** Myofibroma, Myofibromatosis, Pinna, Ear, Histopathology, Pediatric otolaryngology, Case report, Keloid, Hypertrophic

## Abstract

**Background:**

Myofibromas are rare mesenchymal tumors with a predilection for the head, neck, and oral cavity. Primarily affecting infants and young children, these tumors typically manifest as superficial painless nodules. Diagnosis is confirmed through histopathological examination of a biopsy, revealing nodules characterized by spindle cell proliferation. To our knowledge, only two cases of pinna myofibroma have been previously reported in the literature.

**Case presentation:**

Here, we present the case of a three-year-old male who developed a myofibroma of the left auricle following trauma to the area one year earlier. The patient underwent surgical resection without any postoperative complications. The patient later returned with a lesion consistent with hypertrophic scar.

**Conclusions:**

This study aims to provide a comprehensive review of the clinical presentation, histopathologic and immunohistochemical features, and surgical management of this unique case of myofibroma of the pinna.

## Background

Myofibromas are rare benign mesenchymal tumors that typically present as superficial, painless, and rubbery nodules. While they are usually well-circumscribed and mobile, deeper lesions can be fixed. Skin changes such as purplish discoloration, ulceration, and skin atrophy may be present [1–3]. Myofibromas occur in the head and neck in one third of cases, most commonly in the scalp, forehead, and oral cavity [4]. Cases have also been reported in the skull, nose, cheek, ear canal, and pinna. They are more commonly found in males and most often identified in infancy and early childhood; although there are reports in older children and adults [5]. Patients may have a positive family history of myofibroma and both autosomal dominant and recessive forms have been described [2].

Myofibromas are categorized into three types: solitary, multicentric with visceral involvement, and multicentric without visceral involvement. Solitary forms consist of a single nodule and have been shown to regress spontaneously, while multicentric lesions with visceral involvement can be fatal depending on their location [2]. Regression of lesions may be complicated by contraction of surrounding tissue [1]. Factors triggering apoptosis and tumor regression in these lesions are unknown.

On pathology, myofibromas consist of hypocellular lobules with surrounding hypercellular areas, although this zonation of cells is often less apparent in adult lesions compared to the infantile forms [6]. Hypocellular lobules have extensive hyalinization giving a pseudochondroid appearance [3]. The hypercellular areas contain mature spindle-shaped myofibroblasts arranged in bundles or fascicles and small, less differentiated, round cells with scant cytoplasm. Surrounding the lesion are compressed atrophic muscle fibers which can be invaded by proliferating myofibroblasts. Immunostaining shows positivity for alpha smooth muscle actin and vimentin, and negativity for CD34, CD31, 34 glycoprotein, desmin, and s100 [4, 9]. Myofibromas can display features consistent with malignancy such as local invasion, increased cellularity, vascularity, and necrosis; however, metastasis or malignant degeneration has never been reported [3, 9].

Imaging is non-diagnostic and findings can differ depending on the location. Computed tomography typically displays heterogeneous masses with both cystic and solid areas [9]. Ultrasound typically shows hypoechogenicity with or without cystic space. On magnetic resonance imaging, lesions are isodense to muscle on noncontrast T1-weighted images, hyperintense on T2-weighted images, and enhance with gadolinium if increased vascularity is present [10]. Prior case series have shown that when imaging and skeletal surveys were performed, the presence of suspicious lesions ultimately did not change clinical management [9].

Management of myofibromas is not standardized. Conservative management with diagnostic biopsy is typically recommended if the lesion does not affect the function or growth of neighboring vital structures. Many lesions eventually involute, but complete excision can be pursued in patients with visceral lesions, functional impairment, or cosmetic deformity [2]. Chemotherapy has been used previously in lesions that involve vital structures, such as in a case of a spinal cord myofibroma [9]. Recurrence has seldom been reported in the literature, and overall myofibromas have a low recurrence rate. Risk factors for recurrence are location in extremities, age > 5, and previous incomplete excision [11]. Recurrence also partially correlates with the histological subtype, with desmoid-type fibromatosis showing especially high recurrence rates [1]. Many cases have also been reported of biopsies, subtotal resections, and resections with positive margins where lesions have involuted, remained constant, or recurred [9].

Here we report a case of pinna myofibroma in a child that developed after trauma to the area, with formation of a hypertrophic scar after resection of the myofibroma. We describe its clinical features, histopathology, and management of the mass.

## Case presentation

The patient first presented to our Department of Otolaryngology at the age of three for evaluation of a mass on his left auricle. The mass had appeared after a fall from standing onto his ear one year prior to presentation and had been gradually enlarging over this time. The patient’s family denied any pain, drainage, or bleeding from the mass. The patient had no additional lesions on physical exam and had no history of hypertrophic scars or keloids. There was no history of similar lesions in the patient’s family. No other relevant positive symptoms were observed. Despite a course of prescribed antibiotics, there was no reduction in size.

**Fig. 1:**
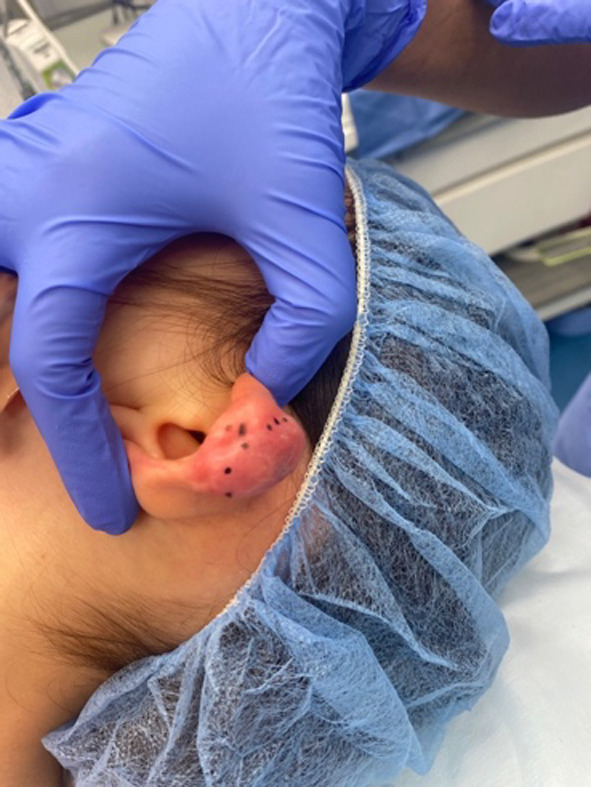
Left auricle lesion in a three-year old male. The lesion is firm and fixed, measuring approximately 1 × 2 cm on the superior aspect of the left helix

Clinical examination showed a firm, fixed lesion measuring approximately 1 × 2 cm on the superior aspect of the left helix. The surrounding skin was non-erythematous. No scars or lesions were noted in the head and neck area. Examination of the tympanic membranes reveal normal clear middle ears. The right ear showed no lesions. The rest of his head and neck exam was unremarkable (Fig. 1).

Given the clinical suspicion of a dermoid cyst, an ultrasound was performed. The ultrasound revealed a well-circumscribed spherical lesion measuring 1.3 cm in diameter. The internal components demonstrated heterogeneity with punctate areas of hyperechogenicity and multiple small areas of shadowing. Doppler evaluation indicated avascularity. This was proposed to be most suggestive of an epidermoid or inclusion cyst with no findings to suggest a soft tissue mass, vascular formation, or retained foreign body. The location was atypical for a congenital cyst of branchial apparatus anomaly. There was minimal hyperechogenicity of the subcutaneous tissues surrounding the lesion, but no doppler hyperemia was present to suggest severe inflammation. The decision was made for surgical excision of the left auricular mass.

Two months after initial presentation to the office, the patient was taken to the operating room for excision of the lesion under general anesthesia. The mass had not changed in size. The mass was carefully dissected from the underlying tissue using sharp, cold dissection. Although it intimated with the surrounding tissue, a plane deep to the lesion allowed it to be separated from healthy tissue and cartilage. It was divided carefully, en-bloc, from the underlying tissue and sent for pathology. No skin or cartilage was taken. The incision was then carefully closed in several layers.

**Fig. 2:**
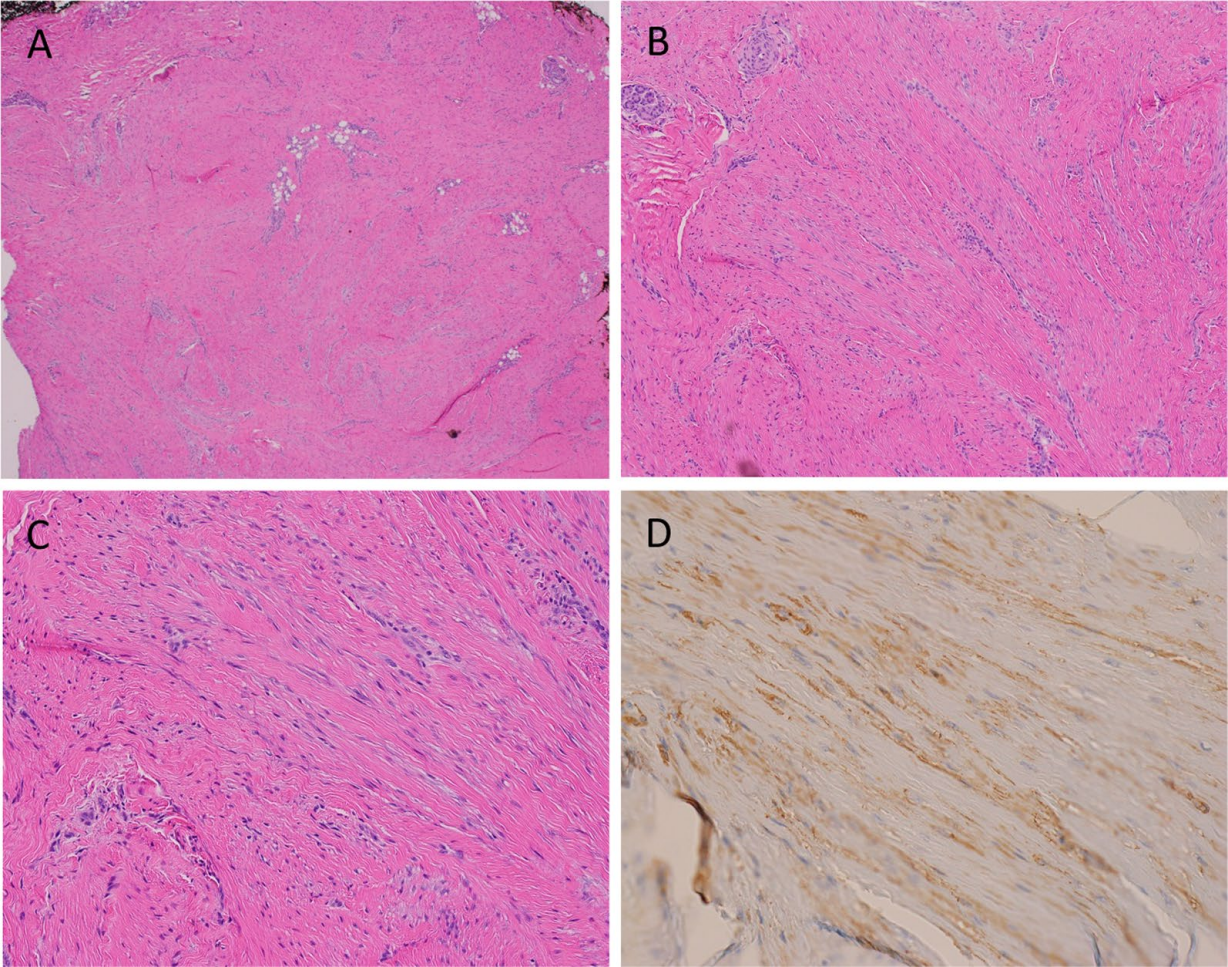
Histopathology of the left ear lesion at **A** 4×, **B** 10×, and **C** 20× magnification. Bland spindle cell and fibrous lesion, suggestive of myofibroma. Microscopic examination reveals a poorly demarcated and low- to moderately-cellular spindle cell and fibrous lesion. Spindle cells are arranged in intersecting and whorled fascicles and appear to entrap adipose tissue and associated skin adnexal structures, but there is no evidence of cytologic atypia or increased mitotic activity. The fibrous stroma is variably sclerotic and focal keloidal type collagen is apparent. Intervening vascular structures including angulated capillary forms and relatively larger and thick-walled arteries are identified; there is patchy vascular congestion, but no red blood cell extravasation is appreciated. **D** By immunoperoxidase staining methods, the spindle cells show patchy weak positivity for SMA indicating myofibroblastic differentiation. A CD34 stain (not shown) is negative in the spindle cells and highlights the component vasculature. The cells are negative for nuclear expression of beta-catenin

A 1.5 × 1.3 × 1.0 cm encapsulated, rubbery mass was sent to pathology. Histopathologic examination revealed a poorly demarcated, low to moderately cellular spindle cell and fibrous lesion suggestive of myofibroma. There was no evidence of cytologic atypia or increased mitotic activity. Immunoperoxidase staining revealed patchy weak positivity for alpha smooth muscle actin (alpha SMA) and were negative for nuclear expression of beta-catenin. A CD34 stain was negative in the spindle cells and highlighted the component vasculature (Fig. 2).

At the 1-week post-operative follow-up, the patient had no postoperative complications, and the incision was noted to be healing appropriately. Nine months following the resection, the parents noted a suspected recurrent mass in the same area. On exam there was a 1.9 × 1 × 0.6 cm cystic lesion at the superior aspect of the auricle. Ultrasound demonstrated a superficial, rounded, well-circumscribed, superficial soft tissue mass. It was heterogenous in echogenicity like the prior lesion; however, it also demonstrated internal vascularity.

The patient was again taken to the operating room for mass excision. Sharp, cold dissection was used to remove the lesion. A plane separating the lesion from underlying healthy tissue was seen and the lesion was removed en-bloc with margin of healthy cartilage given suspected recurrence. A 2.2 × 1.7 × 0.5 cm lesion was sent to pathology. Histopathologic examination revealed a fibroblastic proliferation with focal keloidal type collagen and extravasated RBCs negative for caldesmon, factor XIII, SOX-10, or gene fusions via PCR, suggestive of a nonneoplastic scarring process consistent with hypertrophic scar or keloid.

The patient was seen for a postoperative visit at 1 week and was healing well. The patient has been followed for 4 months with no evidence of recurrent lesion or scar.

## Discussion

Here we present an unusual case of a myofibroma of the pinna that developed after trauma to the area, followed by suspected recurrence after surgical excision later shown to be a hypertrophic scar. Only two prior cases of myofibroma of the pinna have previously been reported in the literature [4, 6], one of which also developed after a history of trauma. Pediatric myofibromas have previously been reported on, notably by Mahajan et al., whose retrospective study included 42 children with myofibroma, 50% of which were located in the head and neck [12]. That study showcased the clinical variability of myofibromas and demonstrated that surgical excision is the preferred treatment.

While trauma has not been proven to be a causative factor in myofibromas, multiple cases have been reported to be associated with trauma [7]. It is hypothesized that this may be due to the role of myofibroblasts in scar formation, as they differentiate from fibroblasts in order to assist with the formation and contraction of granulation tissue. Typically, the myofibroblasts undergo apoptosis when epithelialization is completed, but incomplete apoptosis may lead to fibrosis [8]. Similarly, it is possible that some myofibromas are a result of a self-limited reactive process with incomplete apoptosis. Several cases of myofibroma have also been reported to occur in scar tissue or have preceding injuries that initially resulted in a hematoma, and it is possible that this case represented a similar mechanism of myofibroma formation [5].

The interesting development of a hypertrophic scar following surgical resection is likely related to the location of the initial lesion. In the typical process of wound scarring, the inflammation stage lends way to the formation of granulation tissue, during which myofibroblasts are activated from fibroblasts. These myofibroblasts synthesize and deposit extracellular matrix, as well as possess contractile properties that serve to contract and mature the granulation tissue. The granulation tissue is remodeled as myofibroblasts and other cells undergo apoptosis. When this remodeling process does not occur, the persisting myofibroblasts play a role in pathological scarring, which is characterized by abnormal accumulation of extracellular matrix. Myofibroblasts are sensitive to the mechanical signaling of tension, and so sites of increased tension have a higher likelihood of hypertrophic scar formation. As there is a predilection for development at sites of increased tension, keloids commonly develop on the ears [13]. Because a lack of myofibroblast apoptosis is a major mechanism leading to excessive scarring, it is possible that excess fibroblast stimulation predisposed the patient to develop a myofibroma initially and later a hypertrophic scar in the same location.

We suggest that an area of future study may be to assess the locations of myofibromas of the head and neck, both to study if they may commonly occur in places under tension and to assess whether any factors exist that may predict the occurrence of myofibromas based on location.

## Conclusion

In conclusion, this case represents a unique presentation of a myofibroma of the pinna. Only two prior cases of myofibroma of the pinna have been reported in the literature.

## Data Availability

The data are available from the corresponding author upon request.
